# Prediction of the active compounds and mechanism of Biochanin A in the treatment of Legg-Calvé-Perthes disease based on network pharmacology and molecular docking

**DOI:** 10.1186/s12906-023-04298-w

**Published:** 2024-01-09

**Authors:** Jianhong Liu, Zhirui Hua, Shijie Liao, Boxiang Li, Shengping Tang, Qian Huang, Zhendi Wei, Rongbin Lu, Chengsen Lin, Xiaofei Ding

**Affiliations:** 1 Department of Trauma Orthopedic and Hand Surgery, The First Affiliated Hospital of Guangxi Medical University, 6 Shuangyong Road, Nanning, 530021 Guangxi China; 2 Department of Orthopedics, Minzu Hospital of Guangxi Zhuang Autonomous Region, Nanning, Guangxi China; 3 Guangxi Key Laboratory of Regenerative Medicine, Research Centre for Regenerative Medicine, Guangxi Medical University, Nanning, Guangxi China; 4 Trauma Center, Emergency Department, The First Affiliated Hospital of Guangxi Medical University, 6 Shuangyong Road, Nanning, Guangxi 530021 China

**Keywords:** Network pharmacology, Legg-Calve-Perthes disease, Biochanin A, Endothelial dysfunction

## Abstract

**Background:**

Legg-Calvé-Perthes disease is a special self-limited disease in pediatric orthopedics with a high disability rate and a long-term course, and there is still no clear and effective therapeutic drug in clinic. This study aimed to investigate the potential efficacy of biochanin A, a kind of oxygen-methylated isoflavone compound, in treating Perthes disease based on network pharmacology, molecular docking and in vitro experiments.

**Methods:**

IL-6 was used to stimulate human umbilical vein endothelial cells to construct endothelial cell dysfunction model. We demonstrated whether biochanin A could alleviate endothelial dysfunction through CCK8 assay, immunofluorescence. Targets of biochanin A from pharmMappeer, SWISS, and TargetNet databases were screened. Targets of endothelial dysfunction were obtained from Genecards and OMIM databases. Protein–protein interaction, Gene Ontology, and Kyoto Encyclopedia of Genes and Genomics analyses were used to analyze the potential target and the key pathway of the anti-endothelial dysfunction activity of biochanin A. To validate the potential target-drug interactions, molecular docking and molecular dynamics simulations were performed and the result was proved by western blot.

**Results:**

It was found that biochanin A can promote the expression of ZO-1, reduce the expression of ICAM-1, which means improving endothelial dysfunction. A total of 585 targets of biochanin A from pharmMappeer, SWISS, and TargetNet databases were screened. A total of 10,832 targets of endothelial dysfunction were obtained from Genecards and OMIM databases. A total of 527 overlapping targets of endothelial dysfunction and biochanin A were obtained. AKT1, TNF-α, VCAM1, ICAM1, and NOS3 might be the key targets of the anti-endothelial dysfunction activity of biochanin A, and the key pathways might be PI3K-Akt and TNF signaling pathways. Molecular docking results indicated that the AKT1 and TNF-α had the highest affinity binding with biochanin A.

**Conclusion:**

This study indicates that biochanin A can target AKT1 and TNF-α to alleviate endothelial dysfunction induced by IL-6 in Perthes disease, which provides a theoretical basis for the treatment of Perthes disease by using biochanin A.

**Supplementary Information:**

The online version contains supplementary material available at 10.1186/s12906-023-04298-w.

## Introduction

Legg-Calve-Perthes disease (LCPD) is among the major teratological and disabling diseases of the hip in children. It typically occurs in children aged 4–8 years old (mean, 6.5 years old) [1]. Genetic factors combined with environmental ones characterize the systemic disease known as LCPD [2]. Endothelial dysfunction may lead to thrombosis, increase thrombomodulin levels, and ultimately lead to the occurrence and development of LCPD [3]. Affected children show reduced arterial diameter and function, and the smaller arteries become dysfunctional earlier than the larger ones, which suggests endothelial dysfunction may be a possible pathogenesis of LCPD [4]. In our previous study, circulating endothelial microparticles in LCPD were found to be the reason for endothelial dysfunction and were closely associated with plasma IL-6 concentration [5]. As of this writing, no drug can be effectively used in the clinical treatment of LCPD. Diminishing endothelial dysfunction may be the focus of drug therapy for LCPD.

Biochanin A is a natural oxygen-methylated isoflavone compound. It is a component of many plants, including Spatholobi caulis, soybean, chickpea, red clover, alfalfa and peanut [6]. Biochanin A shows anti-inflammatory [7], anti-tumor [8], anti-microbial [9], antioxidant [10], and neuroprotective activities [11]. This compound decreases lipopolysaccharide-induced inflammation through the inhibition of the expressions of NF-κB and MAPK pathways and the inflammatory factors tumor necrosis factor-α (TNF-α), IL-8, IL-1, vascular cell adhesion molecule-1 (VCAM-1), and intercellular adhesion molecule-1 (ICAM-1). Thus, biochanin A diminishes endothelial dysfunction [12, 13]. Results of studies on animal models indicated that biochanin A induces the inhibition of NF-κB phosphorylation and the reduction of NOS-2, COX-2, inflammatory factor, and PGE2 secretion, thereby antagonizing interleukin-induced inflammation [14]. Biochanin A has potential for use as a therapeutic agent for LCPD.

Natural plant-derived biochanin A’s pharmacological effects are achieved via many targets and pathways. Studies have shown that diseases occur through complex network structures with many diverse phenotypes and targets, and drug research with only one target cannot meet the requirements of disease research [15, 16]. In 2007, Hopkins [17] proposed the idea of "network pharmacology," in which the multi-target action of drugs and the multi-target network of diseases are systematically integrated, and this has become the basis for predicting the drugs that can treat diseases. Thus, a theoretical basis for the study of potential mechanisms of action has been established [18]. Network pharmacology is built on systems biology theories and involves the utilization of high-throughput omics analysis, database search, and computing for exploring drug metabolic properties, toxicity, and function [19, 20]. For analyzing biochanin A’s potential targets and exploring its mechanism of action in LCPD treatment, molecular docking technology and molecular dynamics simulations are used [21].

**Fig. 1:**
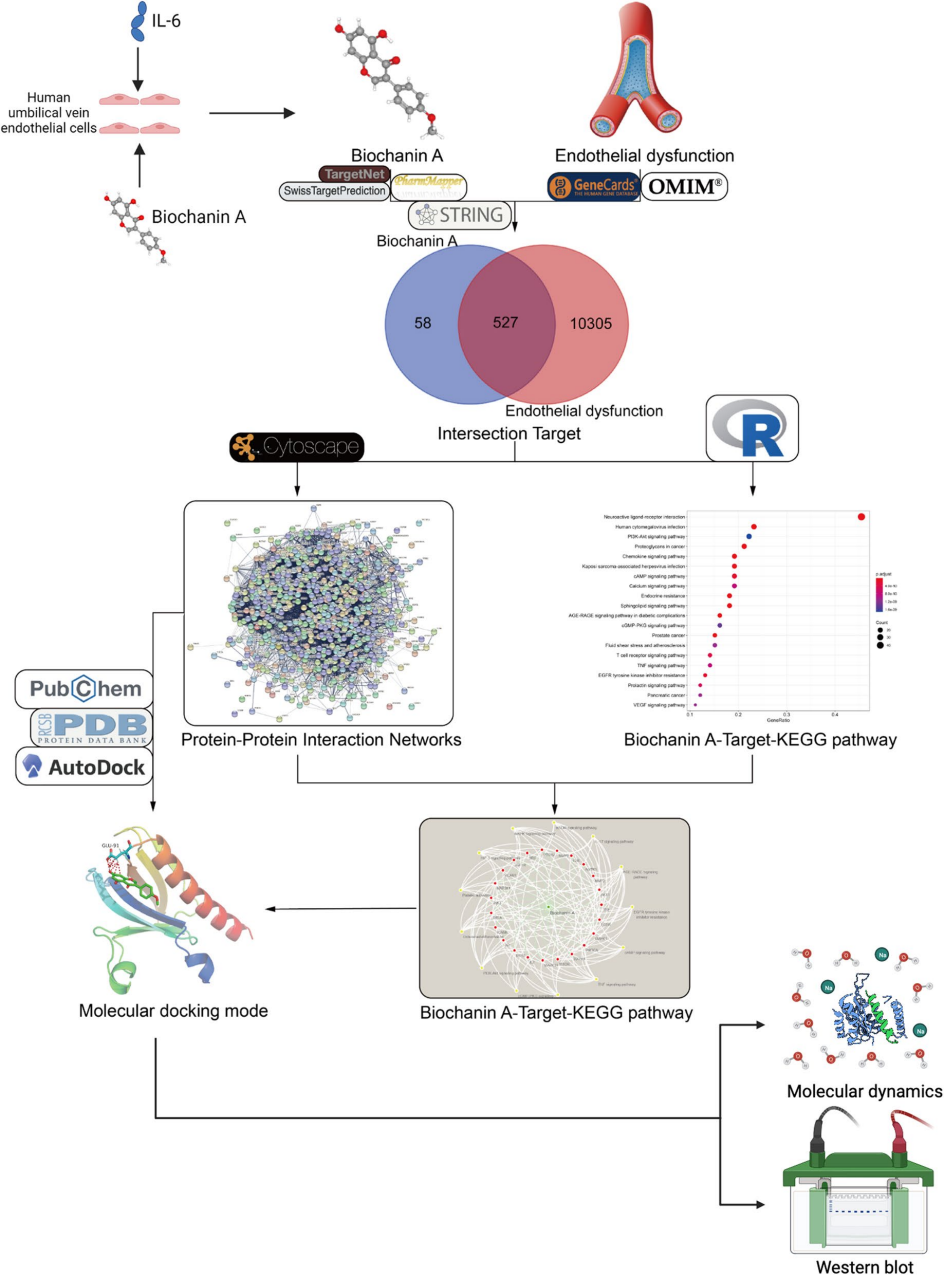
Flow chart of this study. Created with BioRender.com

In conclusion, as shown in the Fig. 1, this study aims to explore the potential target proteins that biochanin A may bind to in LCPD through in vitro experiments and network pharmacology methods, thereby playing a role in improving endothelial dysfunction. In this study, we mainly verified that biochanin A can improve endothelial dysfunction through in vitro experiments. We carried out network pharmacology exploration based on this result, and verified that biochanin A may target protein kinase B (AKT1) and TNF-α through molecular docking technology, western blot. It provides a theoretical basis for the use of biochanin A as a clinical drug for the treatment of LCPD.

## Materials and methods

### Cell culture

Human umbilical vein endothelial cells (HUVECs) were purchased from the Cell Preservation Center of Wuhan University (CCTCC) and cultured in a 37 ℃, 5% CO_2_ incubator with RPIM-1640 complete medium (Gibco, USA) containing 10% fetal bovine serum (EVERY GREEN, China) and 1% penicillin streptomycin mixture (Gibco, USA). When the healing degree of cells in T25 or T75 culture bottles reaches 80%—90%, carry out routine digestion, and then conduct the next experiment on the 6-well plate or 96 well plate.

### CCK8 assay

HUVECs were routinely digested and inoculated into 96 well plates with 3000 cells/well. After cell attachment, different concentrations of IL-6 (0, 1, 10, 100, 1000 pg/mL) (R&D System, USA) or biochanin A (0, 5, 10, 20, 40 μ M) (Desite, China) were added into the plates. After intervention, 10μL CCK8 reagent (HYcezmbio, China) was added to each well and the plate was incubated in the incubator in dark for 1 h. After incubation, the absorbance value (OD) was measured at the wavelength of 450 nm, and the cell activity was calculated.

### Immunofluorescence

After routine digestion, HUVECs were inoculated into 96 well plates with 8000 cells/well. After cell attachment, intervention was divided into the following groups: IL-6 0 pg/mL, IL-6 100 pg/mL, IL-6 100 pg/mL + 5 μM biochanin A、IL-6 100 pg/mL + 10 μM biochanin A. After 24 h of intervention, discard the culture medium, wash it with PBS for three times. Add 4% paraformaldehyde to fix it for 20 min, wash it with PBS for three times after fixation. Add 0.1% Triton-X to penetrate the membrane for 5 min, then add 3% BSA to block for 1 h, wash it with 0.2% BSA twice at the end of blocking. Add primary antibody, and incubate it in a refrigerator at 4 ℃ for 12 h. After incubation, wash it twice with 0.2% BSA. Add secondary antibody, incubate it at room temperature for 2 h. Discard the secondary antibody and wash it twice with 0.2% BSA. Add DAPI to dye it for 5 min. After dyeing, wash twice with 0.2% BSA, and anti-fluorescence quenching agent was added. Observe and take photos under inverted fluorescence microscope. Mean fluorescence intensity was analyzed using ImageJ software.

### Biochanin A targets

The drug target prediction principle is based on ligands’ and proteins’ structural characteristics [22], The following network databases were utilized: Swiss [23] (http://www.swisstargetprediction.ch/); pharmMappeer database [24] (http://lilab-ecust.cn/pharmmapper/); and TargetNet database [25] (http://targetnet.scbdd.com/). The term “Biochanin A” was entered as a search term in each database, and the term “Human” was entered as the study species. For potential drug target retrieval by utilizing the UniProt database (https://www.uniprot.org/uploadlists/), the UniProt ID was converted to Entrez ID through the UniProt database. Then, it was converted to Gene Symbol using DAVID. Biochanin A’s potential targets were identified when the repeated data were excluded.

### Endothelial dysfunction targets

For the determination of the research target databases for common diseases, Genecards data [26] (https://www.genecards.org/) and OMIM database (https://www.omim.org/) were utilized. The related targets of endothelial dysfunction were searched by inputting the term “Endothelial Dysfunction” in these abovementioned databases. DAVID was utilized to convert all the ID into Gene symbol analysis and to remove the repeated targets. Thus, the predicted disease-related targets were obtained.

### Protein–protein interaction (PPI) network

Through the above steps, the possible targets of biochanin A and LCPD were obtained, and their overlapping targets were analyzed through a Venn diagram. They are then imported using the PPI network database String (http://string.db.org/) to map of the PPI (protein protein interaction) network. The term “Biochanin A” was inputted in each database with “Human” as the corresponding study species. Statistical analysis of the interaction score in PPI network was performed by using R software (Version 3.6.3). The top 30 proteins with the highest number of interactions were visually displayed, and the top 100 proteins were used for subsequent analysis.

### Gene Ontology (GO) and Kyoto Encyclopedia of Genes and Genomics (KEGG) pathway analysis

GO and KEGG pathway analyses were used for describing genes and their interrelationships [27]. Gene symbol and Entrez ID data matrix were constructed for the top 100 co-targets of biochanin A and endothelial dysfunction by utilizing the R software (Version 3.6.3) and clusterProfiler package [28] (Version 3.18.1). The database was limited to org.hs.eg. db, and pvalueCutoff and qvalueCutoff = 0.05. Afterward, the GO and KEGG pathway enrichment analyses were performed, and the top 10 bar and top 20 bubble charts were created, respectively. Mapping of the pathway-target network was performed by using Cytoscape software (Version 3.8.0).

### Molecular docking

Biochanin A’s structure SDF format was obtained from the PubChem database and converted into PDB format using Openbabel. This structure was opened by using AutoDockTools1.5.6 [29] and supplemented with hydrogen and charge as ligands. The PDB format of the 3D structures of key target proteins, such as AKT1, TNF-α, nitric oxide synthase 3 (NOS3), VCAM1, and ICAM1, which were screened based on the PPI network, was obtained from the RCSB protein database. The protein structure was separated from water molecules by using the PyMol software [30]. This structure was also opened by using the AutoDockTools1.5.6 software, and hydrogen and charge were added as the receptor. To determine the optional configuration, the AutoDockTools1.5.6 was also used for docking. Binding energy’s lowest conformation was analyzed, and the binding mode was obtained. For the visual operation, PyMol software was utilized.

### Molecular dynamics simulation

Molecular dynamics simulations [31] were conducted using Gromacs 2020.6 software for a duration of 50 ns. Prior to the simulation, the system underwent energy optimization and energy minimization (canonical ensemble, NVT) utilizing the mdrun command and employing the fastest descent method. The starting step size was set to 0.01 nm with a maximum allowable force value of 1000 kJ/mol•nm. After the optimization of the system energy was completed, a 100 ps NVT simulation (isothermal isosteric) was performed on the system at a fixed volume and constant heating rate, causing the system temperature to slowly rise from 0 K to 310.15 K, further unifying the distribution of solvent molecules in the solvent box. Subsequently, a 100 ps NPT ensemble simulation (isobaric isobaric) was conducted using a Berendsen constant pressure device to perform pressure equilibrium on the solvent composite system, raising the system pressure to 1 bar. During Molecular Dynamics simulation, all hydrogen bonds were constrained using LINCS algorithm with an integration step of 2 fs. Electrostatic interactions were calculated using Particle-mesh Ewald (PME) method with a cutoff value of 1.2 nm. The nonbonded interaction cutoff was set at 10 A and updated every 10 steps. The simulated trajectories were periodically eliminated, followed by subsequent analysis including Root-mean-square deviation (RMSD), Root Mean Square Fluctuation (RMSF), radius of gyration (Rg) and hydrogen bond analyses. Finally, the initial and final conformations obtained from simulations were compared.

### Western blot assay

After routine digestion, HUVECs were inoculated into 6-well plates at a rate of 3 × 10^5^ cells/well. After cell attachment, the intervention groups were as follows: IL-6 0 pg/mL, IL-6 100 pg/mL, IL-6 100 pg/mL + 5 μM biochanin A, IL-6 100 pg/mL + 10 μM biochanin A. After 24 h of intervention, the medium was discarded, and 120μL cell lysate was added to each well. The cells were scraped off with the cell scraper and incubated on ice for 20 min. At the end of the incubation, the lysate was collected in a 1.5 mL EP tube and centrifuged at 12000 rpm/min for 20 min, and the supernatant was aspirated into another 1.5 mL EP tube. After extraction, the protein samples were separated by 12% sodium dodecyl sulfate–polyacrylamide gel electrophoresis, and then transferred to PVDF membrane (Biosharp, China) for blotting. The membrane was washed three times with 1 × TBST, blocked by adding fast blocking solution (Beyotime, China) for 15 min, and washed three times with 1 × TBST at the end of blocking. The corresponding primary antibodies, namely TNF-α (Signalway Antibody, China), AKT1 (Signalway Antibody, China) and p-AKT1 (Signalway Antibody, China), were added, and incubated at 4℃ for 12-14 h. At the end of the incubation, the membrane was washed with 1 × TBST for three times and the secondary antibody was added. The membrane was incubated on a shaker at room temperature for 1 h, and then washed with 1 × TBST for three times. The image of the membrane was displayed with Image Quant LAS 4000 system and analyzed with ImageJ software.

### Statistical analysis

All data analysis was carried out in SPSS 26.0, and the mapping software used GraphPad 7.0, AI and other software for comprehensive processing. One-way ANOVA was used to analyze the differences between groups. And the post-test used in this study is Tukey test. When the p value was less than 0.05, it was considered that the difference was statistically significant.

## Results

### Biochanin A alleviates IL-6-induced endothelial dysfunction

**Fig. 2:**
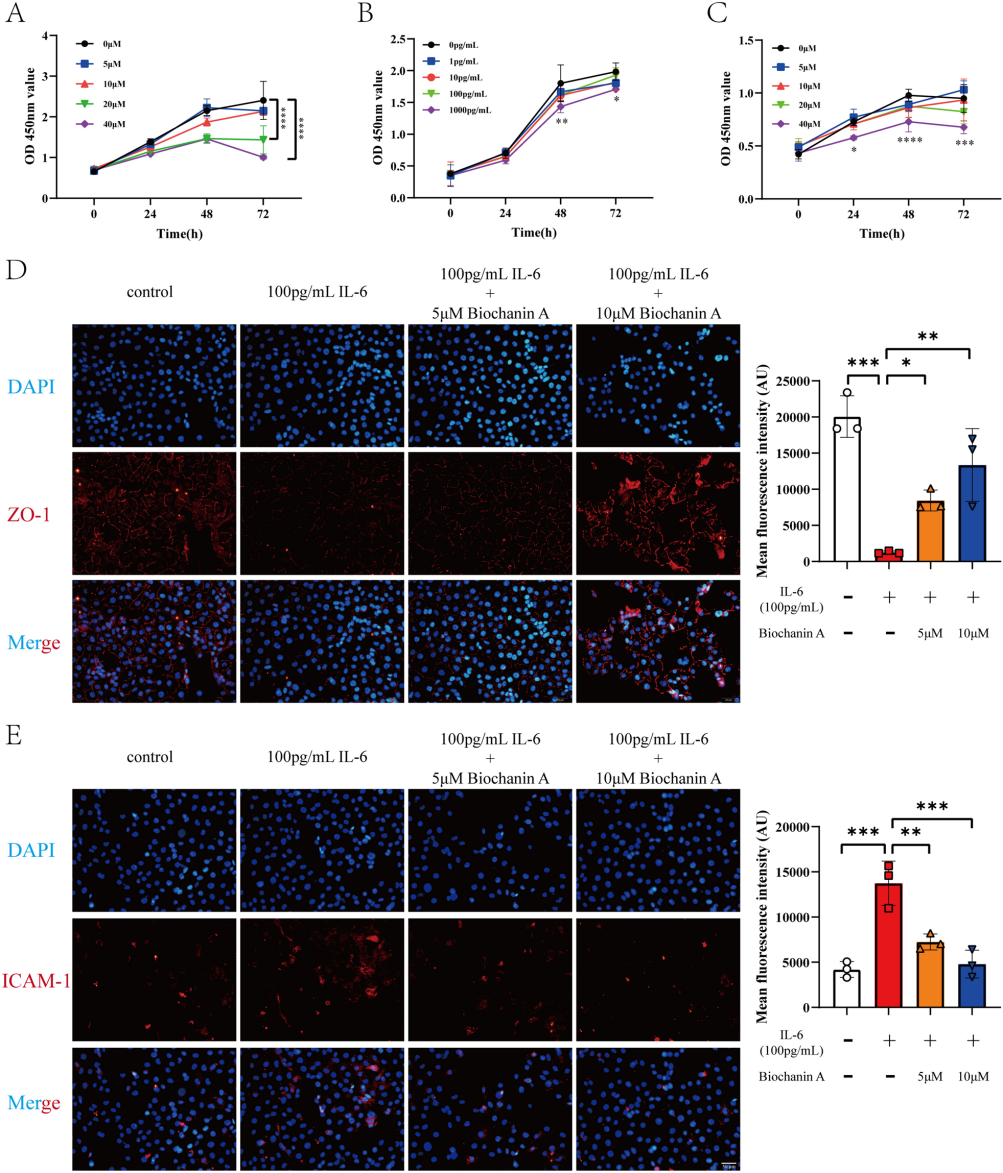
Biochanin A alleviates endothelial dysfunction induced by IL-6. **A** CCK8 result of different concentration of biochanin A showed that 0–10 μM biochanin A did not have toxic effect on HUVECs. (*n* = 3) **B** CCK8 result of different concentration of IL-6 showed that 0–100 pg/mL IL-6 did not have toxic effect on HUVECs. (*n* = 3) **C** CCK8 result of 100 pg/mL IL-6 combined with 0–10 μM biochanin A showed that the intervention did not have toxic effect on HUVECs. (*n* = 3) **D** The result of immunofluorescence showed that 100 pg/mL IL-6 caused the decreased expression of ZO-1 and biochanin A restored the expression of ZO-1. (*n* = 3) **E** The result of immunofluorescence showed that 100 pg/mL IL-6 caused the increased expression of ICAM-1 and biochanin A decreased the expression of ICAM-1. (*n* = 3) *p < 0.05, **p < 0.01, ***p < 0.001, ****p < 0.0001. #p < 0.05, 20 μM biochanin A compared with 0 μM biochanin A

After the intervention of HUVECs with different concentrations of IL-6 and biochanin A, the optimal intervention concentration was determined by CCK8 assay. The results showed that 0-100 pg/mL IL-6 and 0-10 μM biochanin A did not affect the cell viability. When 100 pg/mL IL-6 was combined with 0-10 μM biochanin A, there was no toxic effect on cells (Fig. 2 A-C). Based on this, we further performed immunofluorescence and found that the expression of ICAM-1 increased and ZO-1 decreased after IL-6 intervention, indicating endothelial dysfunction at this time. However, when biochanin A was added, ICAM-1 expression decreased and ZO-1 expression recovered (Fig. 2D, E). These results indicated that biochanin A alleviated IL-6-induced endothelial dysfunction.

### Intersection targets of biochanin A and endothelial dysfunction

**Fig. 3:**
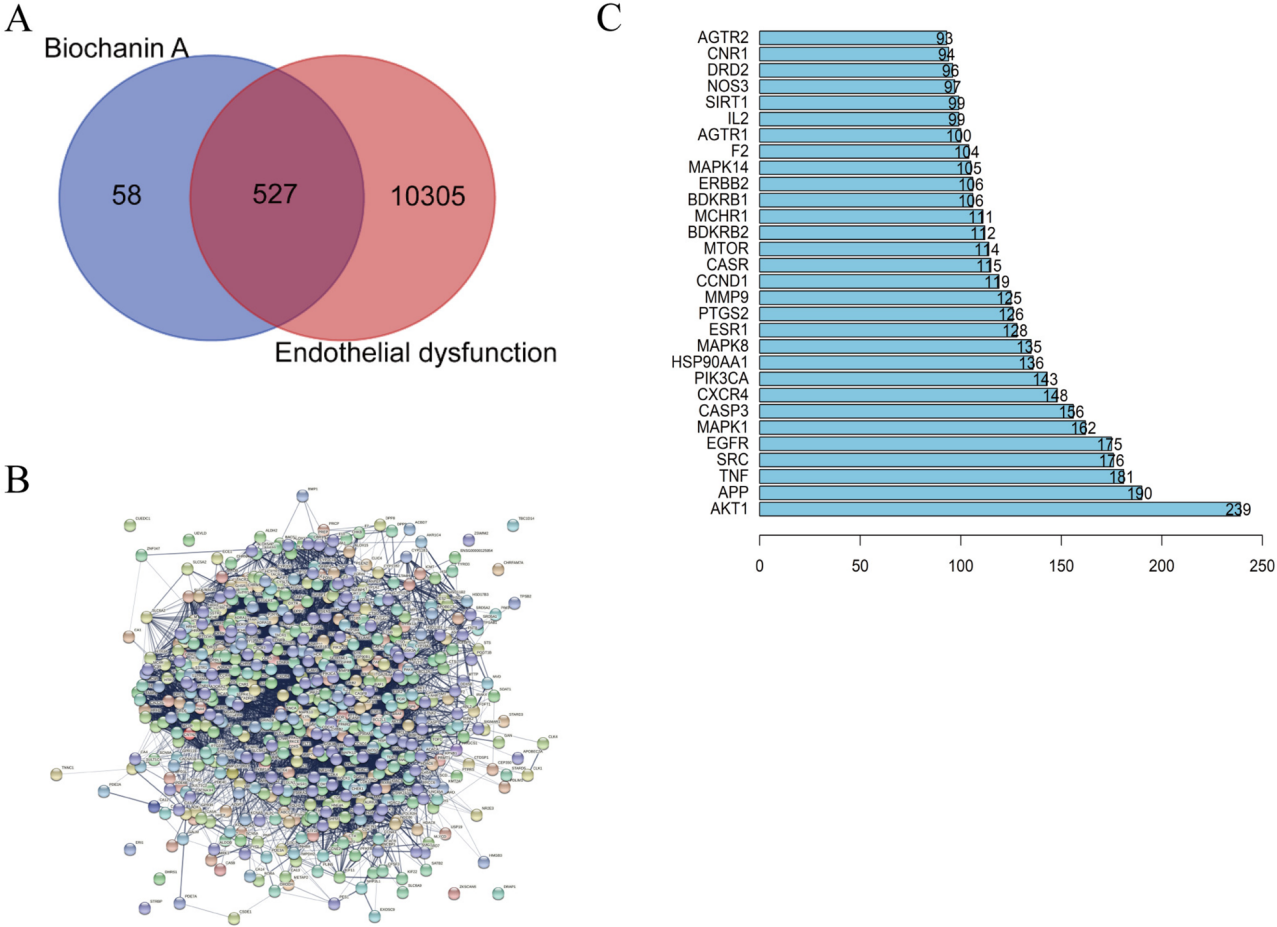
Biochanin A and prediction of key therapeutic targets for endothelial dysfunction. **A** Venn diagram of the target gene of biochanin A and endothelial dysfunction; **B** PPI network of the co-targets of biochanin A and endothelial dysfunction; **C** The top 30 proteins in PPI protein interaction with other proteins

The pharmMappeer, SWISS, and TargetNet databases were searched for potential target genes of biochanin A. Duplicate data were excluded, and 585 target genes of biochanin A were obtained. A total of 10,832 target genes of endothelial dysfunction were obtained by consulting disease-related databases, such as Genecards and OMIM, by using the term "Endothelial Dysfunction" after duplicate data removal. The Venn diagram was constructed, and it revealed that 527 targets intersected with one another (Fig. 3A).

### PPI network of targets

For drawing the PPI network, details of the protein interaction obtained from the Venn diagram were imported into the PPI network database String (Fig. 3B). R software was used for the further evaluation of the PPI analysis results. Figure 3C shows the top 30 proteins that interacted the most with other proteins in the PPI network. AKT1 was the protein with the highest number of interactions, thereby suggesting that it plays a critical role in biochanin A’s prevention and treatment of endothelial dysfunction. The top 100 proteins in the PPI network that showed the highest number of interactions with other proteins were included in the subsequent analysis.

### GO enrichment analysis

**Fig. 4:**
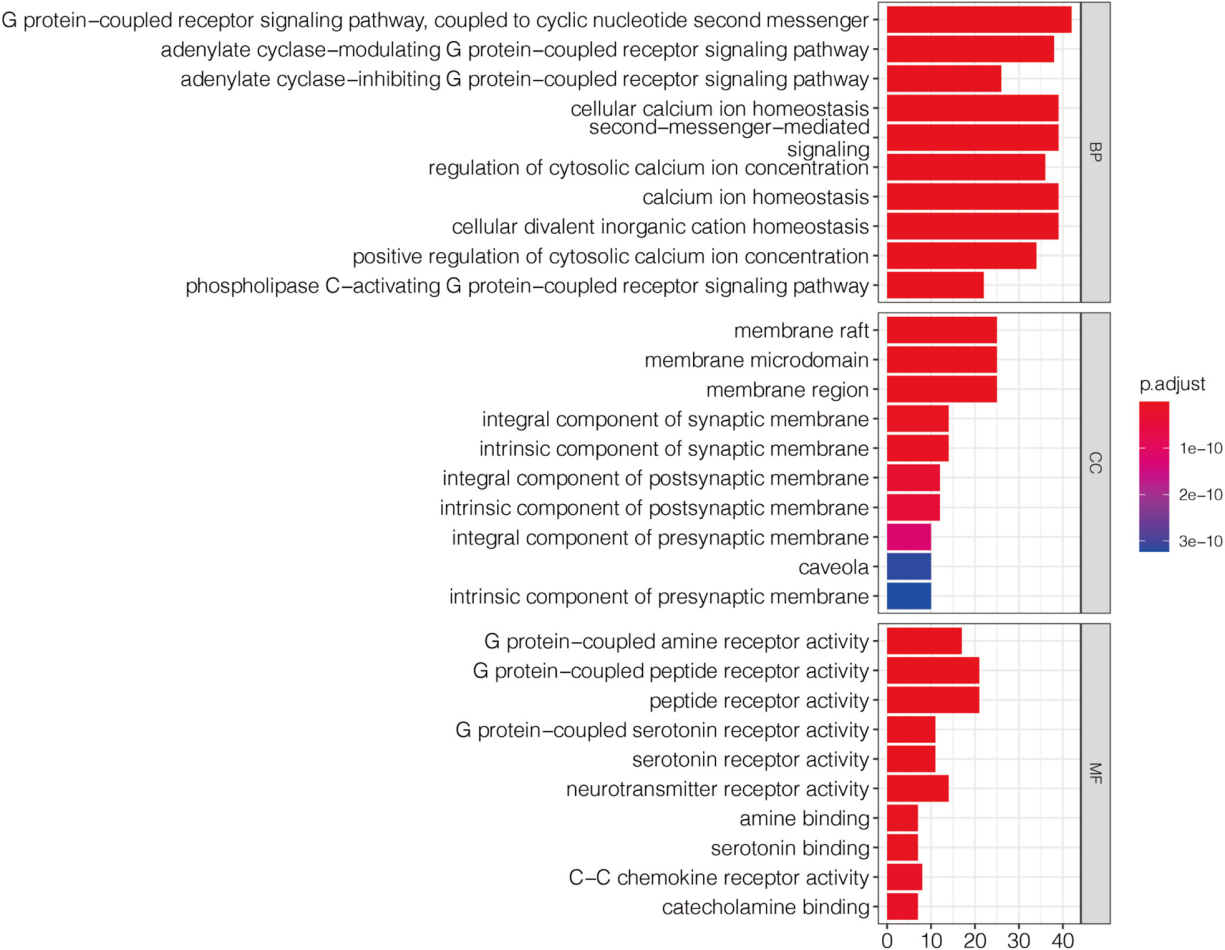
GO enrichment analysis. The top 100 key targets in PPI network were selected for GO enrichment analysis

GO enrichment analysis process includes the cellular component (CC), molecular function (MF), and biological process (BP). A total of 527 potential targets were involved in the following: “G protein − coupled receptor signaling pathway, coupled to cyclic nucleotide second messenger”, “adenylate cyclase − modulating G protein − coupled receptor signaling pathway”, “adenylate cyclase − inhibiting G protein − coupled receptor signaling pathway”, “cellular calcium homeostasis” and “second − messenger − mediated signaling”. The MFs had relationships with “G protein − coupled amine receptor activity”, “G protein − coupled peptide receptor activity”, “peptide receptor activity”, “G protein − coupled serotonin receptor activity”, and “neurotransmitter receptor activity”. According to CC analysis, the protein’s proportion in the membrane microregion was higher. The 10 BPs are listed in Supplementary Table I. This finding indicated that biochanin A regulated many different MFs and participated in varied BPs to produce effects (Fig. 4).

### KEGG pathway analysis

**Fig. 5:**
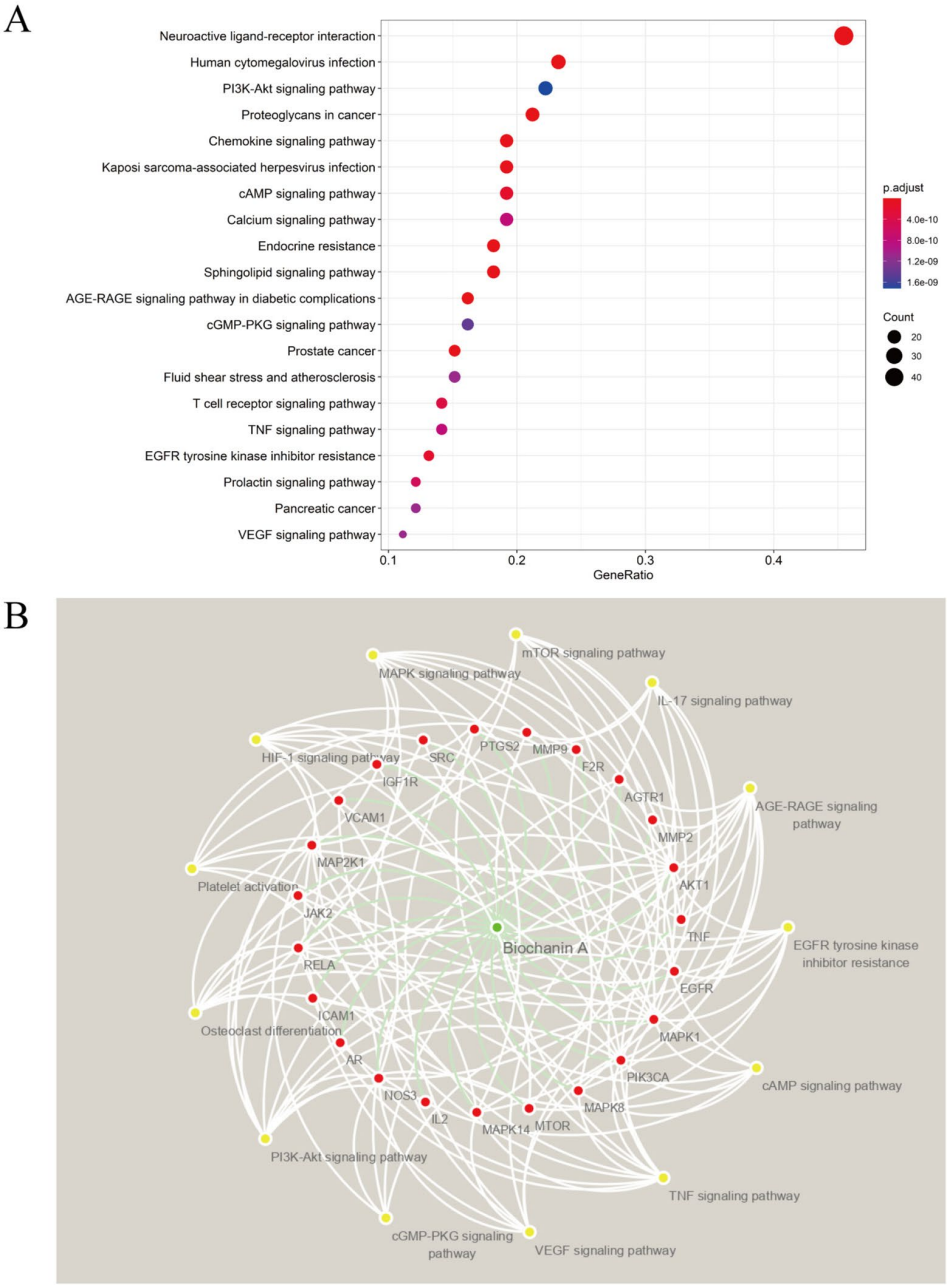
KEGG pathway enrichment analysis

An enrichment analysis of KEGG pathway was conducted for the intersection targets, and the top 20 significant pathways (*P* < 0.05) were obtained for visual display. Among these, the neuroactive ligand-receptor interaction was the signal pathway with the highest score and that involved the highest number of targets (Fig. 5A). The 20 pathways are listed in Supplementary Table II. Biochanin A possibly exerts pharmacological effects through cell biological phenotypes, as follows: cell apoptosis, cell migration, cell survival, vascular smooth muscle function, vascular tension, prostaglandin production and inflammation. Studies showed maps of signal pathways related to endothelial dysfunction and the key targets contained in each pathway. Biochanin A may pass through AKT1, TNF, EGFR, MAPK1, PIK3CA, MAPK8, MTOR, MAPK14, IL2, NOS3, AR, ICAM1, RELA, JAK2, MAP2K1, VCAM1, IGF1R, SRC, PTGS2, MMP9, F2R, AGTR1, and MMP2. Endothelial function was treated through the regulation of EGFR tyrosine kinase inhibitor resistance and the following: AGE-RAGE signaling pathway, cAMP signaling pathway, TNF signaling pathway, VEGF signaling pathway, GMP-PKG signaling pathway, PI3K-Akt signaling pathway, HIF-1 signaling pathway, MAPK signaling pathway, mTOR signaling pathway, and other pathway obstacles (Fig. 5B). The Supplementary Figures 1 and 2 shows the pathway-target network diagrams of PI3K-Akt and TNF signaling respectively [32–34].

The top 100 key targets in the PPI network were selected for KEGG gene pathway enrichment analysis. A: Bubble map of the KEGG gene pathway enrichment analysis. B: Map of signal pathways that are associated with endothelial dysfunction and the key targets contained in each pathway. Green, red, and yellow circles represent drugs, key targets, and related pathways, respectively.

### Molecular docking

**Fig. 6:**
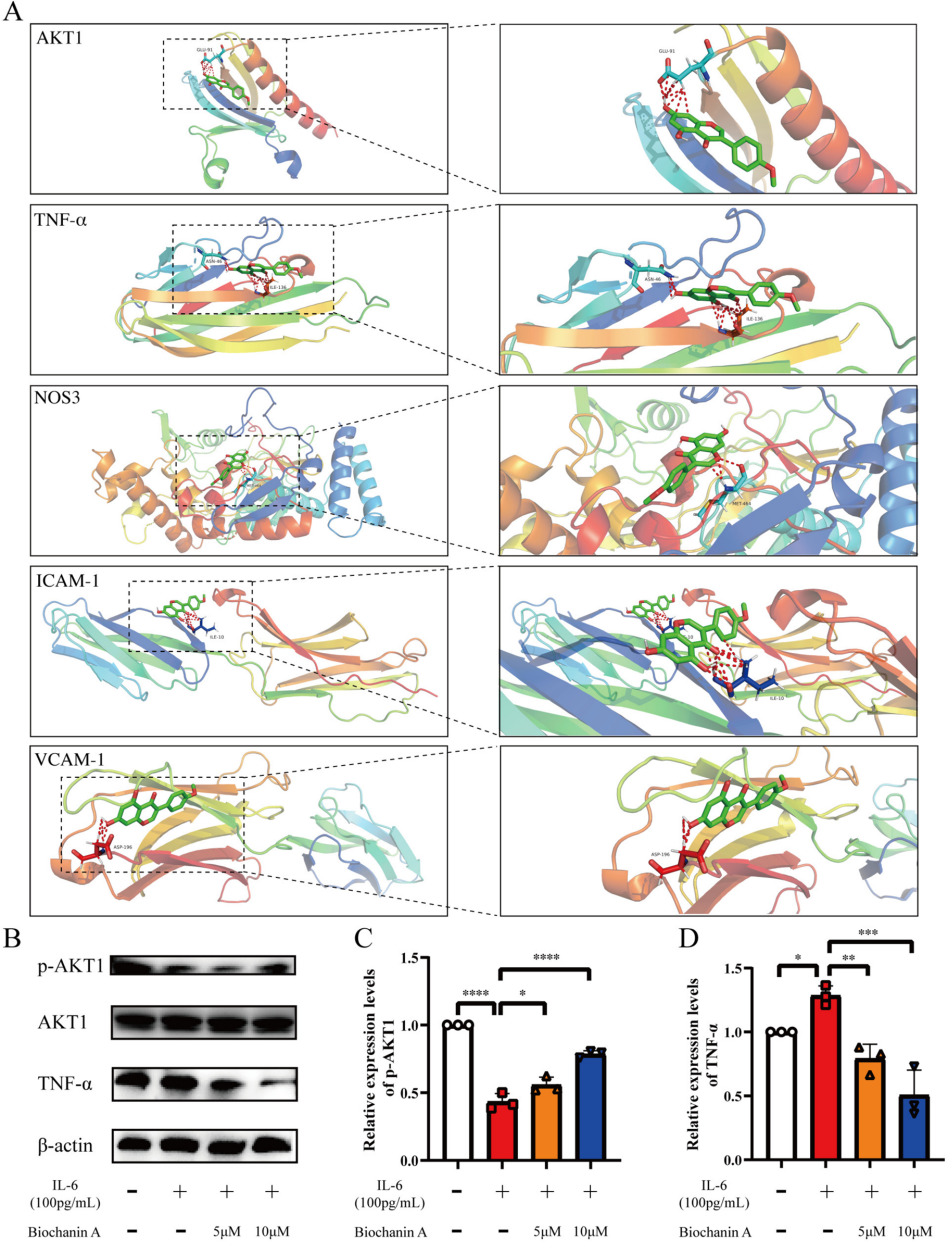
Molecular docking. **A** The result of molecular docking. **B** The result of Western blot showed that biochanin A reduced the expression of TNF-α and increased the phosphorylation level of AKT1. Full-length blots are presented in Supplementary Figure 3. (*n* = 3) **C**, **D** Statistical analysis of the phosphorylation level of AKT1 (**C**) and the expression level of TNF-α (**D**). *p < 0.05, **p < 0.01, ***p < 0.001, ****p < 0.0001

Many studies of molecular docking have shown that stable complexes are formed when the free binding energy is negative. The lower the binding energy required for the ligand to bind to the receptor, the more likely docking is to occur. We tested the following potential target proteins (Fig. 6A): AKT1(PDB: 1unq), TNF-α (PDB: 2az5), ICAM1 (PDB: 1iam), VCAM1 (PDB: 1vsc) and NOS3(PDB: 4d1p). They are key targets in the network of interactions, and the docking binding energy results are -5.19 kcal/mol, -4.61 kcal/mol, -3.87 kcal/mol, -3.54 kcal/mol and -4.731 kcal/mol, respectively. Biochanin A binds to Glu-91 of AKT1 protein and ASN-46 and ILe-136 of TNF-α protein, respectively, with minimal and negative binding energy. This finding indicated that the ligand had a good binding activity with the target protein and resulted in stable binding. Other targets could also bind biochanin A to form stable complexes. Figure 6A presents the docking results, which indicated that biochanin A can closely bind to the predicted target. This is probably the main reason for the reduction of endothelial function. Subsequently, we performed western blot experiment. The result showed that biochanin A could increase the expression level of p-AKT1 and decrease the expression level of TNF-α in HUVECs intervened by IL-6, which was a further validation of the result of molecular docking (Fig. 6B-D).

### Molecular dynamics simulation

**Fig. 7:**
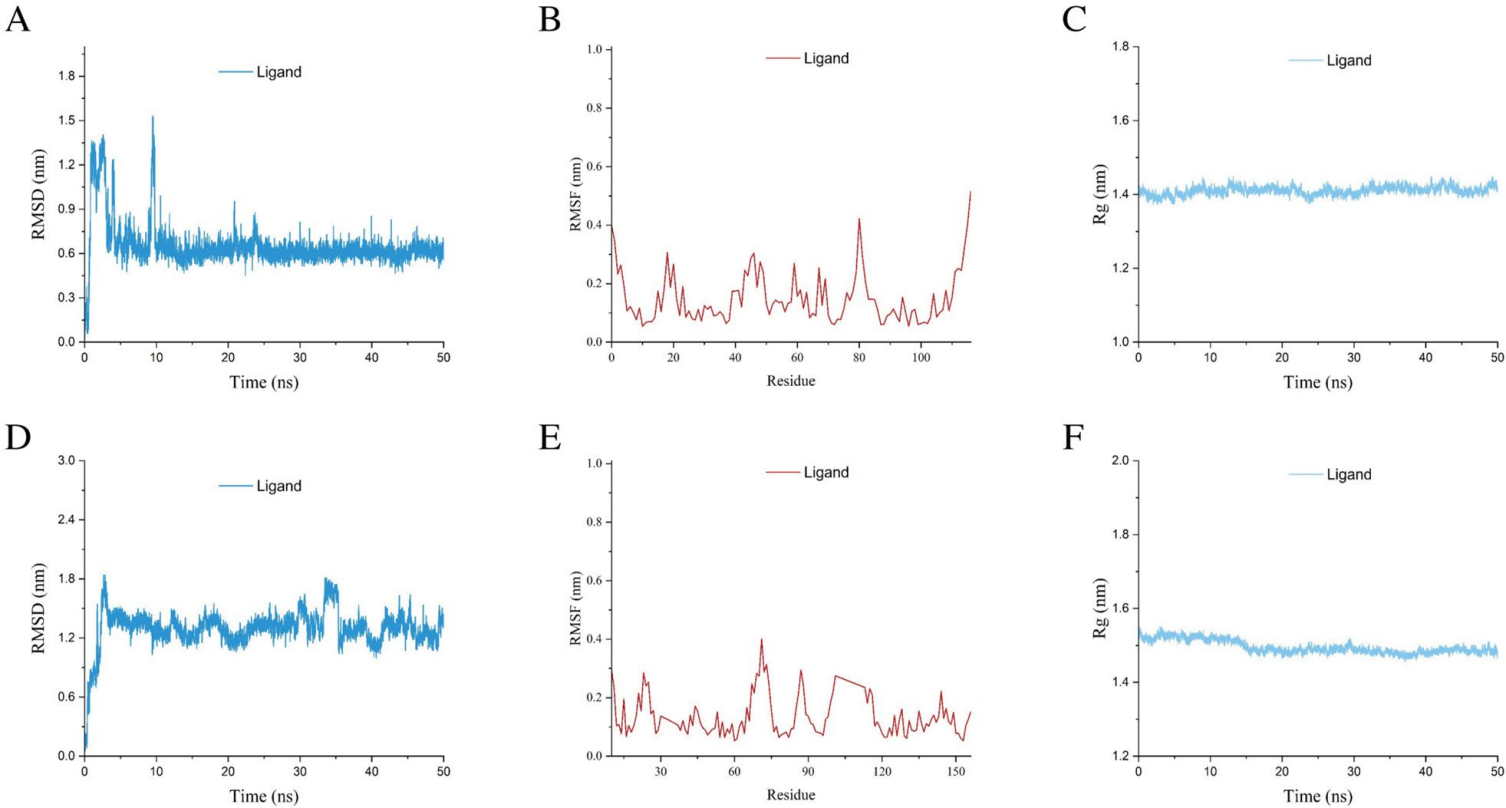
Molecular Dynamics Simulation. **A** RMSD Analysis of biochanin A-AKT1. **B** RMSF Analysis of biochanin A-AKT1. **C**: Rg of biochanin A-AKT1. D: RMSD Analysis of biochanin A-TNF-α. **E** RMSF Analysis of biochanin A-TNF-α. **F** Rg of biochanin A-TNF-α

Analyze the simulated trajectory and evaluate the RMSD, RMSF, and Rg. RMSD serves as the standard for evaluating system stability. Based on the analysis of the RMSD diagram and the movement trajectory of small molecule protein complexes, it could be seen that although the RMSD curve of small molecules fluctuated during the simulation process, there was never a phenomenon of detachment from the protein binding cavity, such as AKT1 protein and TNF- α. After 10 ns and 4 ns, the RMSD curve gradually stabilized, indicating the formation of stable complexes between proteins and small molecules during this simulated period (Fig. 7A, D). The RMSF value of the protein is relatively low, indicating that the protein is relatively stable during the simulation process, and this sampling is relatively reliable (Fig. 7B, E). During the entire simulation process, there was no significant disturbance of the protein side chain relative to the main chain, and it remained relatively stable (Fig. 7C, F). Therefore, the overall results demonstrated good stability between the small-molecular ligand–protein acceptor complex. (For details on Hbond, molecular docking model, and Align, please refer to Supplementary Fig. 4).

## Discussion

LCPD is among the most common hip disabling diseases in children. Endothelial dysfunction is possibly a key part of LCPD pathogenesis. We previously showed that plasma endothelial microparticles phenotype reflected endothelial dysfunction phenotype in children with LCPD and that Perthes-microparticles could lead to endothelial dysfunction. Moreover, IL-6 can promote endothelial dysfunction and stimulate endothelial cell-secreted endothelial microparticles to induce endothelial dysfunction and inhibit angiogenesis [5]. It has been 100 years since the discovery of LCPD, but there is still no effective drug for its clinical treatment. The anti-inflammatory and anti-endothelial dysfunction effects of biochanin A have been previously described [12, 35]. Biochanin A has potential for use as an effective drug treatment of LCPD. However, its specific mechanism needs to be clarified.

We demonstrated in vitro that the expression of ICAM-1 was increased and the expression of ZO-1 was decreased after IL-6 induced endothelial dysfunction. Kim et al. have demonstrated that IL-6 is significantly elevated in the synovial fluid of children with LCPD. Our study also found that IL-6 is also significantly elevated in the plasma of children with LCPD, suggesting that IL-6 may be a key pro-inflammatory factor in LCPD [36]. Therefore, we speculate whether biochanin A can reduce the expression of IL-6, inhibit inflammation, improve IL-6 induced endothelial dysfunction, and promote femoral head vascular reconstruction. ICAM-1 is a cell surface glycoprotein that binds leukocyte adhesion and endothelial function. Under inflammatory stimulation, activated endothelial cells increase the expression of ICAM-1. ICAM-1 recruitment of excessive white blood cells can exacerbate the inflammatory process and, in many cases, exacerbate tissue damage [37] High expression of ICAM-1 can lead to endothelial dysfunction and inflammatory processes [38]. Increased ICAM-1 levels may serve as a molecular marker for the development of atherosclerosis and clinical coronary heart disease [37]. ZO-1 plays a significant role in maintaining the tight connection between the blood–brain barrier and endothelial cells [39, 40], and it is also one of the most commonly used markers in corneal endothelial cells [41], which suggests that it can be used as a marker of endothelial dysfunction. No study has shown that ICAM-1 and ZO-1 can be used clinically as a diagnostic marker of LCPD, but in our in vitro study, the expression of ICAM-1 was decreased and ZO-1 was restored after intervention with biochanin A. Therefore, ICAM-1 and ZO-1 may be used to assist the clinical diagnosis of LCPD. A total of 527 intersecting targets of biochanin A and endothelial dysfunction were identified through network pharmacology. Figure 3B and C present the PPI results. AKT1 is the target with the highest number of interactions. TNF, VCAM-1, ICAM-1, and NOS3 are also key targets in the PPI network. AKT, which is also protein kinase B, affects the expression and/or activity of various angiogenic factors. The AKT subtypes (AKT1, AKT2, and AKT3) were previously identified as potential therapeutic targets for ischemic injury [42]. AKT1 is a major subtype in vascular cells [43] that promotes angiogenesis through phosphorylation and by regulating important downstream targets, e.g., NOS3 [44]. NOS3 is endothelial nitric oxide synthase. It produces NO in endothelial cells, and its metabolic changes usually cause endothelial dysfunction [45]. Progression of endothelial dysfunction is associated with the increase in the expressions of chemokines, cellular adhesion molecules, and cytokines [46], such as VCAM-1, and systemic inflammatory markers, such as TNF-α, among others [47]. VCAM-1 is a known mediator of eosinophils and monocytes. It combines with vascular endothelial cell adhesion factors and is highly expressed in endothelial and smooth muscle cells. VCAM-1 is involved in inflammation and progress, thereby contributing to endothelial dysfunction. Reduction of VCAM-1 gene expression can reduce vascular inflammation and endothelial dysfunction [48, 49]. TNF-α is a cytokine that increases the expression of inflammatory factors. It induces the expressions of ICAM-1 and VCAM-1, helps reduce the interaction among endothelial cells, and accelerates the progression of inflammation and endothelial dysfunction [50]. It has been proved to be the key target of formononetin against atherosclerosis by improving endothelial dysfunction [51].

Results of GO biological function analysis and KEGG pathway enrichment analysis indicated that biochanin A is involved in the treatment of endothelial dysfunction through many different biological processes and signaling pathways. The KEGG pathway enrichment analysis involves PI3K-Akt, TNF, and AGE-RAGE signaling pathways. When induced, the pharmacological effects may eventually be evident in various cell biological phenotypes, such as inflammation, vascular smooth muscle function, prostaglandin production, and vascular tension, as well as cell migration, survival, and apoptosis. Literature suggests that biochanin A alleviates endothelial dysfunction through a complex network structure that has many targets and pathways. The molecular docking between small molecules and coding proteins of biochanin A and its key targets has been verified in the present study, thereby providing a strong foundation for the future validation and transformation of clinical results.

Network pharmacology was used to construct a drug-target-critical pathway diagram (Fig. 4B). AKT1 is involved in PI3K-Akt, VEGF, and TNF signaling pathways and is a key target in the complex drug–disease interaction network. PI3K-Akt signaling pathway activation in endothelial cells regulates endothelial cell survival and migration, as well as capillary-like structure formation, which are key steps in angiogenesis [52]. LEE indicated that PI3K-AKT1-NO signaling pathway is needed for vascular tone regulation and adaptive vascular remodeling [53]. In Somanath et al.’s study, results of gene knockout mouse experiments showed that AKT1 deficiency affects VEGF expression, wound angiogenesis, and subsequent vascular system maturation [54]. VEGF in turn acts on endothelial cell surface receptors, thereby triggering downstream signals, such as those of PI3K-Akt and NOS3, to promote angiogenesis [55]. TNF signaling pathway has a relationship with immune response and inflammation It mediates pathways by activating various receptors, such as MAPK and NF-κB signaling through the TNFR1 receptor and PI3K-Akt signaling through the TNFR2 receptor [56, 57]. VCAM1 and ICAM1 are potential targets of biochanin A that are involved in AGE-RAGE signaling pathways and are associated with endothelial dysfunction. Advanced glycation end products (AGEs) interact with their cellular receptors (RAGE) to activate NF-κB by activating nicotinamide adenine dinucleotide phosphate (NADPH) oxidase. TNF-α, cell adhesion molecule, tissue factor, cytokine, and monocyte chemotactic protein-1 (McP-1) expressions were induced [58, 59]. Therefore, slowing AGE-RAGE signaling pathway expression can alleviate endothelial dysfunction [60]. In network pharmacology, NOS3, as a potential target of biochanin A, is involved in AGE-RAGE, VEGF, GMP-PKG, PI3K-Akt, platelet activation, and HIF-1 signaling pathways. NOS3 plays a role in the pathophysiology of femoral head necrosis through vasodilation, angiogenesis, platelet activation, and other mechanisms. NOS3, as an enzyme complex, is a key molecule involved in the occurrence of endothelial dysfunction. It promotes nitric oxide (NO). Oxidative stress can lead to the dysregulation of NOS3 and endothelial dysfunction [61, 62]. The 27-bp VNTR polymorphism in intron 4 of NOS3 gene and the G894T polymorphism in exon 7 in children with LCPD were possibly associated with the disease’s etiology, as we mentioned previously [63].

Of course, the clinical transformation and application of biochanin A is still not mature at present. Although some studies have shown that its side effects are less than other anti-inflammatory drugs, biochanin A still has shortcomings such as poor absorption, rapid systemic clearance, and accelerated metabolism, which can reduce its bioavailability. This may cause certain limitations in clinical treatment [64]. Meanwhile, our current research still has certain limitations, such as the lack of clinical proof of the diagnostic efficacy of ICAM-1 and ZO-1, and the specific mechanism of action of biochanin A is not yet clear. In the future, we will further explore the mechanism by which biochanin A can improve endothelial dysfunction by binding to its target proteins AKT1 and TNF-α, to provide theoretical support for the possibility of biochanin A as a clinical therapeutic drug.

## Conclusion

We have demonstrated through in vitro experiments that IL-6 intervention in HUVECs can cause changes in the expression levels of endothelial dysfunction markers ICAM-1 and ZO-1 ——an increase in ICAM-1 expression and a decrease in ZO-1 expression. However, the use of biochanin A improved IL-6 induced endothelial dysfunction. Biochanin A possibly prevents endothelial dysfunction via a complex network structure with multiple targets and pathways, among which AKT1, TNF-α, NOS3, ICAM-1, and VCAM-1 may be the key targets, according to network pharmacological analysis results. The most probable binding targets of biochanin A were AKT1 and TNF-α according to molecular docking screening results. This study introduced new ideas and potential therapeutic drugs for the treatment of LCPD and provided a basis for clinical transformation.

### Supplementary Information

**Additional file 1.**

## Data Availability

The datasets generated during and/or analyzed during the current study are available from the corresponding author on reasonable request. The datasets generated and/or analysed during the current study are available in the Worldwide Protein Data Bank (wwPDB) repository, RCSB PDB: Homepage.

## Abbreviations

LCPD: Legg-Calve-Perthes disease
IL-6: Interleukin-6
PPI: Protein–protein interaction
GO: Gene Ontology
KEGG: Kyoto Encyclopedia of Genes and Genomics
ZO-1: Zonula occludens-1
ICAM1: ICAM-1 Intercellular adhesion molecule 1
AKT1: Protein kinase B
TNF-α: Tumor necrosis factor-α
VCAM1: VCAM-1 Vascular cell adhesion molecule 1
NOS3: Nitric oxide synthase 3
PI3K: Phosphoinositide-3-kinase
NF-κB: Nuclear factor kappa-B
MAPK: Mitogen-activated protein kinase
