# Supplementary material for: Prediction of the active compounds and mechanism of Biochanin A in the treatment of Legg-Calvé-Perthes disease based on network pharmacology and molecular docking
Source: BMC Complement Med Ther. 2024 Jan 9;24:26. doi: 10.1186/s12906-023-04298-w (PMC10775507; doi:10.1186/s12906-023-04298-w)
Supplement: Supplementary file 1 — Additional file 1. [file 12906_2023_4298_MOESM1_ESM.docx]

**Supplementary table**

**Supplementary table I. Biological processes of potential target genes based on GO enrichment analysis.**

| GO ID | Biological function | Genes | P-value |
| --- | --- | --- | --- |
| GO:0007187 | G protein-coupled receptor signaling pathway, coupled to cyclic nucleotide second messenger | CASR, MCHR1, DRD2, CNR1, AGTR2, OPRM1, ADRA2A, DRD4, HTR1A, ADRA2B, ADRA2C, OPRL1, P2RY12, S1PR1, CCR1, OPRK1, OPRD1, DRD3, ADORA1, CCR3, CHRM2, HTR5A, ADRA1B, GCGR, HTR2A, PTGER3, HTR1B, GRM2, HRH3, CNR2, GRM4, S1PR2, HTR2C, PTGDR2, S1PR3, ADRB2, HTR1D, S1PR4, ADRA1A, CHRM1, EDNRA, HTR1E | 5.45E-52 |
| GO:0007188 | adenylate cyclase-modulating G protein-coupled receptor signaling pathway | CASR, MCHR1, DRD2, CNR1, OPRM1, ADRA2A, DRD4, HTR1A, ADRA2B, ADRA2C, OPRL1, P2RY12, S1PR1, OPRK1, OPRD1, DRD3, ADORA1, CCR3, CHRM2, HTR5A, ADRA1B, GCGR, PTGER3, HTR1B, GRM2, HRH3, CNR2, GRM4, S1PR2, PTGDR2, S1PR3, ADRB2, HTR1D, S1PR4, ADRA1A, CHRM1, EDNRA, HTR1E | 3.92E-47 |
| GO:0007193 | adenylate cyclase-inhibiting G protein-coupled receptor signaling pathway | CASR, MCHR1, DRD2, OPRM1, ADRA2A, DRD4, HTR1A, OPRL1, P2RY12, S1PR1, OPRK1, OPRD1, DRD3, ADORA1, CHRM2, HTR5A, HTR1B, GRM2, HRH3, GRM4, PTGDR2, S1PR3, HTR1D, CHRM1, EDNRA, HTR1E | 1.12E-39 |
| GO:0006874 | cellular calcium ion homeostasis | APP, CXCR4, ESR1, CASR, BDKRB2, MCHR1, BDKRB1, F2, AGTR1, IL2, DRD2, CNR1, CCR5, PTPRC, CCR2, CXCR2, DRD4, OPRL1, JAK2, S1PR1, CCR1, F2R, FYN, CXCR3, DRD3, ADORA1, CCR3, CXCR1, ADRA1B, LYN, HTR2A, PTGER3, HTR1B, LCK, HTR2C, S1PR3, S1PR4, ADRA1A, EDNRA | 4.12E-37 |
| GO:0019932 | second-messenger-mediated signaling | TNF, EGFR, CXCR4, MTOR, AGTR1, NOS3, DRD2, CNR1, AGTR2, CCR5, OPRM1, PTPRC, CCR2, CXCR2, ADRA2A, KDR, ADRA2B, ADRA2C, OPRL1, P2RY12, S1PR1, CCR1, CXCR3, DRD3, CCR3, CXCR1, HTR5A, ADRA1B, GCGR, PTGER3, CNR2, S1PR2, HTR2C, PTGDR2, S1PR3, ADRB2, S1PR4, VCAM1, ADRA1A | 4.12E-37 |
| GO:0051480 | regulation of cytosolic calcium ion concentration | CXCR4, ESR1, BDKRB2, MCHR1, BDKRB1, F2, AGTR1, IL2, DRD2, CNR1, CCR5, PTPRC, CCR2, CXCR2, OPRL1, JAK2, S1PR1, CCR1, F2R, FYN, CXCR3, DRD3, ADORA1, CCR3, CXCR1, ADRA1B, LYN, HTR2A, PTGER3, HTR1B, LCK, HTR2C, S1PR3, S1PR4, ADRA1A, EDNRA | 1.06E-36 |
| GO:0055074 | calcium ion homeostasis | APP, CXCR4, ESR1, CASR, BDKRB2, MCHR1, BDKRB1, F2, AGTR1, IL2, DRD2, CNR1, CCR5, PTPRC, CCR2, CXCR2, DRD4, OPRL1, JAK2, S1PR1, CCR1, F2R, FYN, CXCR3, DRD3, ADORA1, CCR3, CXCR1, ADRA1B, LYN, HTR2A, PTGER3, HTR1B, LCK, HTR2C, S1PR3, S1PR4, ADRA1A, EDNRA | 1.14E-36 |
| GO:0072503 | cellular divalent inorganic cation homeostasis | APP, CXCR4, ESR1, CASR, BDKRB2, MCHR1, BDKRB1, F2, AGTR1, IL2, DRD2, CNR1, CCR5, PTPRC, CCR2, CXCR2, DRD4, OPRL1, JAK2, S1PR1, CCR1, F2R, FYN, CXCR3, DRD3, ADORA1, CCR3, CXCR1, ADRA1B, LYN, HTR2A, PTGER3, HTR1B, LCK, HTR2C, S1PR3, S1PR4, ADRA1A, EDNRA | 8.05E-36 |
| GO:0007204 | positive regulation of cytosolic calcium ion concentration | CXCR4, ESR1, BDKRB2, MCHR1, BDKRB1, F2, AGTR1, IL2, DRD2, CNR1, CCR5, PTPRC, CCR2, CXCR2, OPRL1, JAK2, S1PR1, CCR1, F2R, FYN, CXCR3, DRD3, CCR3, CXCR1, ADRA1B, LYN, HTR2A, PTGER3, LCK, HTR2C, S1PR3, S1PR4, ADRA1A, EDNRA | 2.86E-35 |
| GO:0007200 | phospholipase C-activating G protein-coupled receptor signaling pathway | ESR1, CASR, F2, AGTR1, DRD2, OPRM1, CXCR2, ADRA2A, OPRL1, P2RY12, S1PR1, F2R, OPRK1, OPRD1, DRD3, CHRM2, ADRA1B, HTR2A, PTGER3, HTR2C, ADRA1A, CHRM1 | 1.00E-29 |

**Supplementary table II. Potential target gene function based on Kyoto Encyclopedia of Genes and Genomes pathway analysis.**

| KEGG ID | Pathway | Pathway genes | P‐value |
| --- | --- | --- | --- |
| hsa04080 | Neuroactive ligand-receptor interaction | BDKRB2, MCHR1, BDKRB1, F2, AGTR1, DRD2, CNR1, AGTR2, OPRM1, ADRA2A, DRD4, HTR1A, ADRA2B, ADRA2C, OPRL1, S1PR1, F2R, OPRK1, OPRD1, DRD3, ADORA1, ADORA3, CHRM2, HTR5A, ADRA1B, GCGR, HTR2A, PTGER3, HTR1B, GRM2, HRH3, NR3C1, CNR2, GRM4, S1PR2, HTR2C, S1PR3, ADRB2, HTR1D, NPY5R, S1PR4, ADRA1A, CHRM1, EDNRA, HTR1E | 3.08E-36 |
| hsa05163 | Human cytomegalovirus infection | AKT1, TNF, SRC, EGFR, MAPK1, CASP3, CXCR4, PIK3CA, PTGS2, CCND1, MTOR, MAPK14, CCR5, GRB2, CXCR2, RELA, CCR1, MDM2, CCR3, MAP2K1, PTGER3, CASP8, PTK2 | 1.55E-15 |
| hsa04151 | PI3K-Akt signaling pathway | AKT1, EGFR, MAPK1, PIK3CA, HSP90AA1, CCND1, MTOR, ERBB2, IL2, NOS3, BCL2L1, GRB2, KDR, RELA, JAK2, F2R, MDM2, CHRM2, MAP2K1, PTK2, CHRM1, IGF1R | 1.69E-10 |
| hsa05205 | Proteoglycans in cancer | AKT1, TNF, SRC, EGFR, MAPK1, CASP3, PIK3CA, ESR1, MMP9, CCND1, MTOR, ERBB2, MAPK14, MMP2, GRB2, CDC42, KDR, MDM2, MAP2K1, PTK2, IGF1R | 3.01E-14 |
| hsa04020 | Calcium signaling pathway | EGFR, CXCR4, BDKRB2, BDKRB1, ERBB2, AGTR1, NOS3, KDR, F2R, CHRM2, HTR5A, ADRA1B, HTR2A, PTGER3, HTR2C, ADRB2, ADRA1A, CHRM1, EDNRA | 5.84E-11 |
| hsa05167 | Kaposi sarcoma-associated herpesvirus infection | AKT1, SRC, MAPK1, CASP3, PIK3CA, MAPK8, PTGS2, CCND1, MTOR, MAPK14, CCR5, ICAM1, RELA, JAK2, CCR1, CCR3, LYN, MAP2K1, CASP8 | 1.21E-12 |
| hsa04024 | cAMP signaling pathway | AKT1, MAPK1, PIK3CA, MAPK8, DRD2, HTR1A, RELA, F2R, ADORA1, CHRM2, MAP2K1, PTGER3, HTR1B, ADRB2, HTR1D, CHRM1, EDNRA, HCAR2, HTR1E | 9.14E-12 |
| hsa04062 | Chemokine signaling pathway | AKT1, SRC, MAPK1, CXCR4, PIK3CA, CCR5, GRB2, CCR2, CDC42, CXCR2, RELA, JAK2, CCR1, CXCR3, CCR3, CXCR1, LYN, MAP2K1, PTK2 | 1.11E-12 |
| hsa01522 | Endocrine resistance | AKT1, SRC, EGFR, MAPK1, PIK3CA, MAPK8, ESR1, MMP9, CCND1, MTOR, ERBB2, MAPK14, MMP2, GRB2, MDM2, MAP2K1, PTK2, IGF1R | 6.94E-17 |
| hsa04071 | Sphingolipid signaling pathway | AKT1, TNF, MAPK1, PIK3CA, MAPK8, BDKRB2, MAPK14, NOS3, RELA, S1PR1, FYN, OPRD1, ADORA1, ADORA3, MAP2K1, S1PR2, S1PR3, S1PR4 | 2.54E-15 |
| hsa04933 | AGE-RAGE signaling pathway in diabetic complications | AKT1, TNF, MAPK1, CASP3, PIK3CA, MAPK8, CCND1, MAPK14, AGTR1, NOS3, MMP2, CDC42, ICAM1, RELA, JAK2, VCAM1 | 4.14E-14 |
| hsa04022 | cGMP-PKG signaling pathway | AKT1, MAPK1, BDKRB2, AGTR1, NOS3, ADRA2A, ADRA2B, ADRA2C, OPRD1, ADORA1, ADORA3, ADRA1B, MAP2K1, ADRB2, ADRA1A, EDNRA | 1.33E-10 |
| hsa05215 | Prostate cancer | AKT1, EGFR, MAPK1, PIK3CA, HSP90AA1, MMP9, CCND1, MTOR, ERBB2, GRB2, AR, RELA, MDM2, MAP2K1, IGF1R | 4.64E-13 |
| hsa05418 | Fluid shear stress and atherosclerosis | AKT1, TNF, SRC, PIK3CA, HSP90AA1, MAPK8, MMP9, MAPK14, NOS3, MMP2, KDR, ICAM1, RELA, PTK2, VCAM1 | 9.67E-11 |
| hsa04660 | T cell receptor signaling pathway | AKT1, TNF, MAPK1, PIK3CA, MAPK8, MAPK14, IL2, GRB2, PTPRC, CDC42, RELA, FYN, MAP2K1, LCK | 2.08E-11 |
| hsa04668 | TNF signaling pathway | AKT1, TNF, MAPK1, CASP3, PIK3CA, MAPK8, PTGS2, MMP9, MAPK14, ICAM1, RELA, MAP2K1, CASP8, VCAM1 | 5.81E-11 |
| hsa01521 | EGFR tyrosine kinase inhibitor resistance | AKT1, SRC, EGFR, MAPK1, PIK3CA, MTOR, ERBB2, BCL2L1, GRB2, KDR, JAK2, MAP2K1, IGF1R | 8.22E-12 |
| hsa04917 | Prolactin signaling pathway | AKT1, SRC, MAPK1, PIK3CA, MAPK8, ESR1, CCND1, MAPK14, GRB2, RELA, JAK2, MAP2K1 | 3.33E-11 |
| hsa05212 | Pancreatic cancer | AKT1, EGFR, MAPK1, PIK3CA, MAPK8, CCND1, MTOR, ERBB2, BCL2L1, CDC42, RELA, MAP2K1 | 9.13E-11 |
| hsa04370 | VEGF signaling pathway | AKT1, SRC, MAPK1, PIK3CA, PTGS2, MAPK14, NOS3, CDC42, KDR, MAP2K1, PTK2 | 8.87E-11 |

**Supplementary figure**


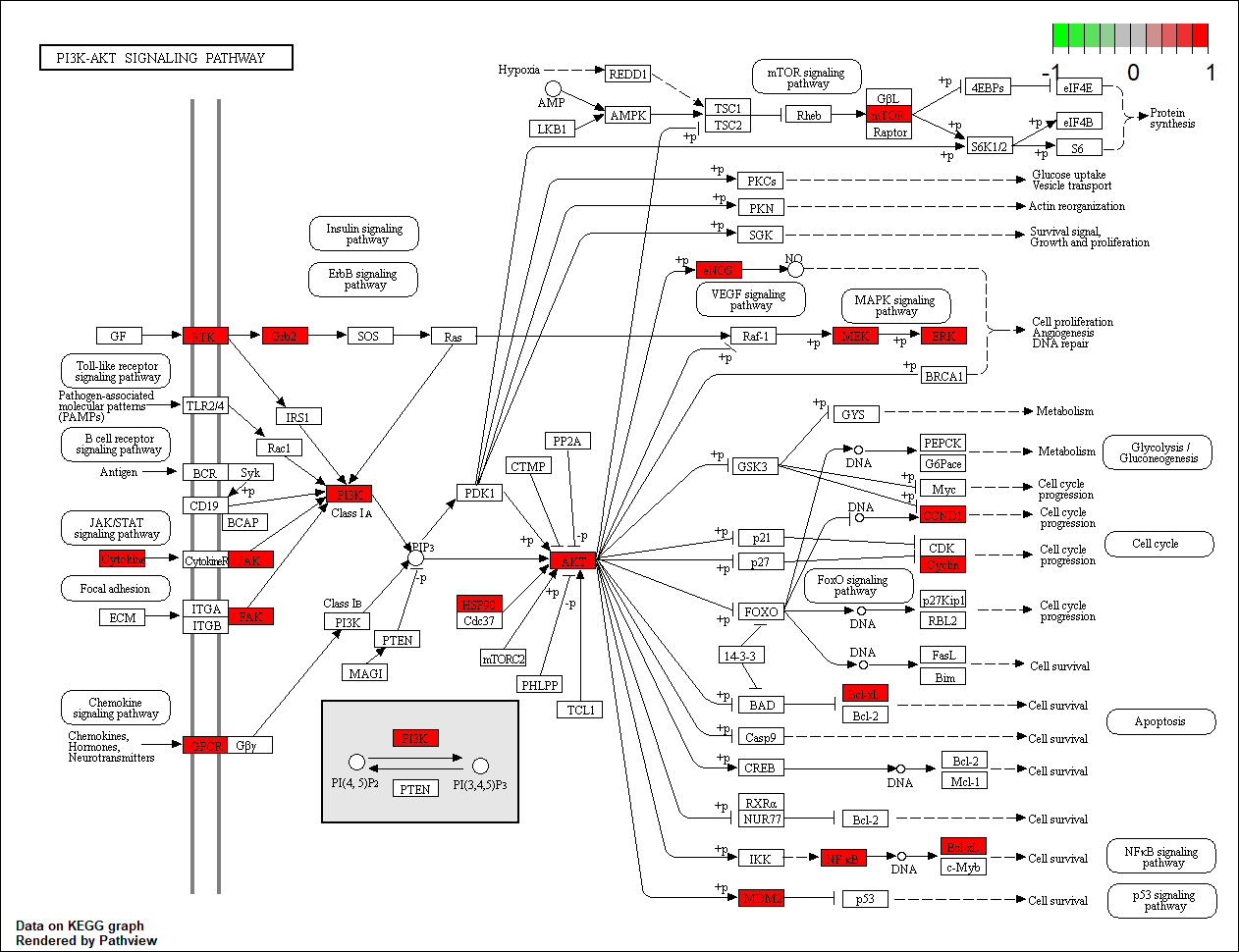


**Supplementary fig. 1 Pi3k-akt signaling pathway - target network diagram**
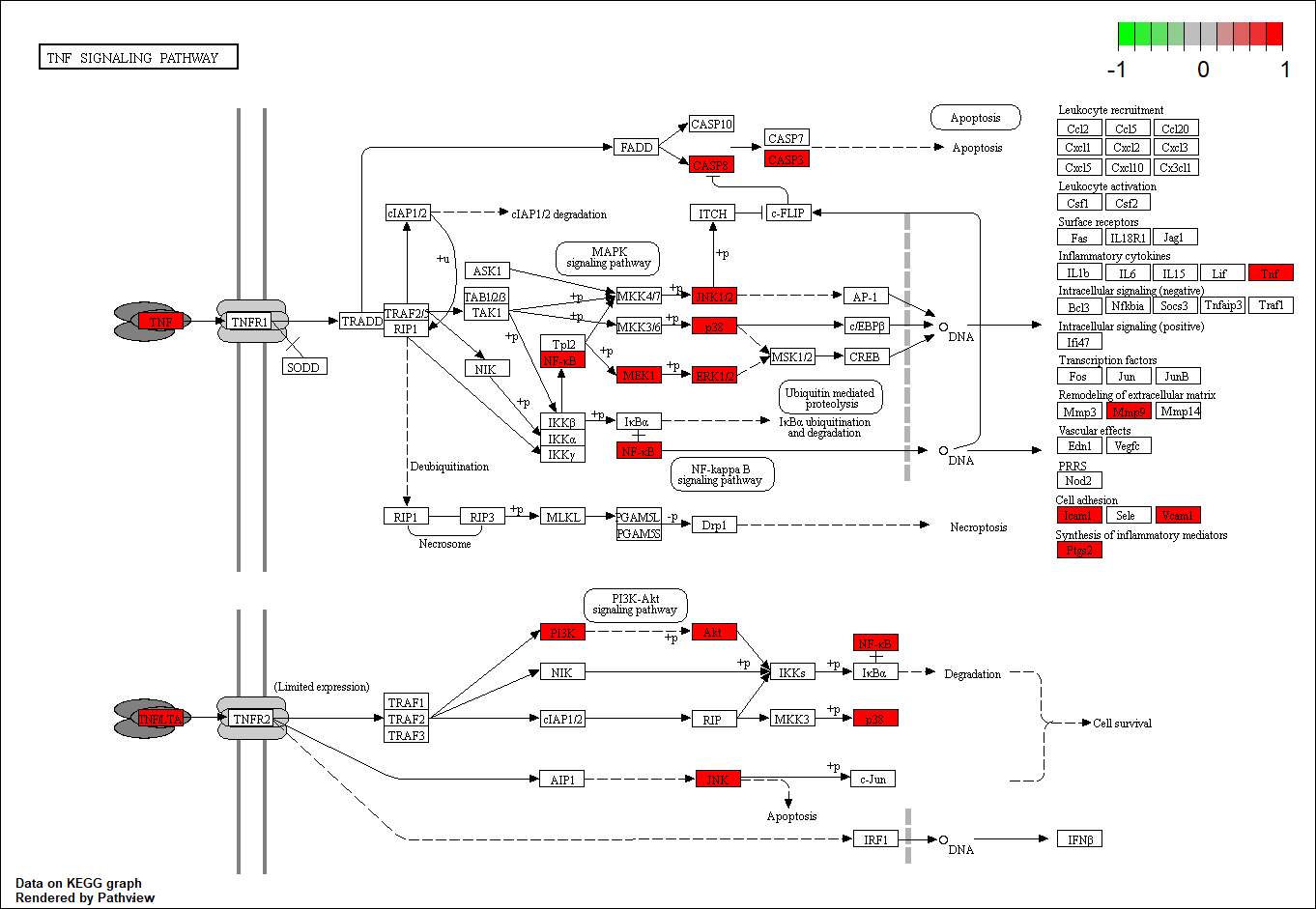


**Supplementary fig. 2 TNF signaling pathway - target network diagram**


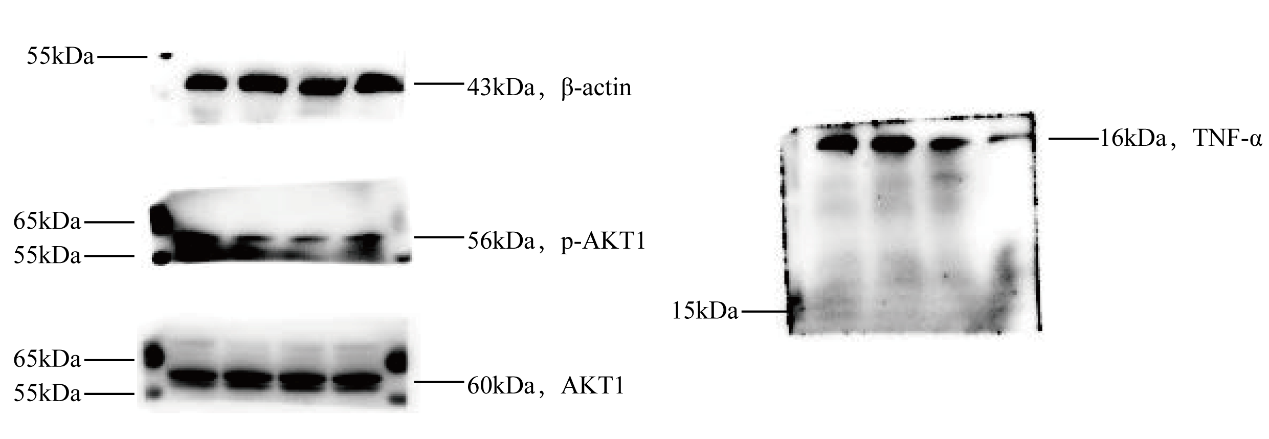


**Supplementary fig. 3 Full-length blots of the Western blot bands of Figure 5. B.**


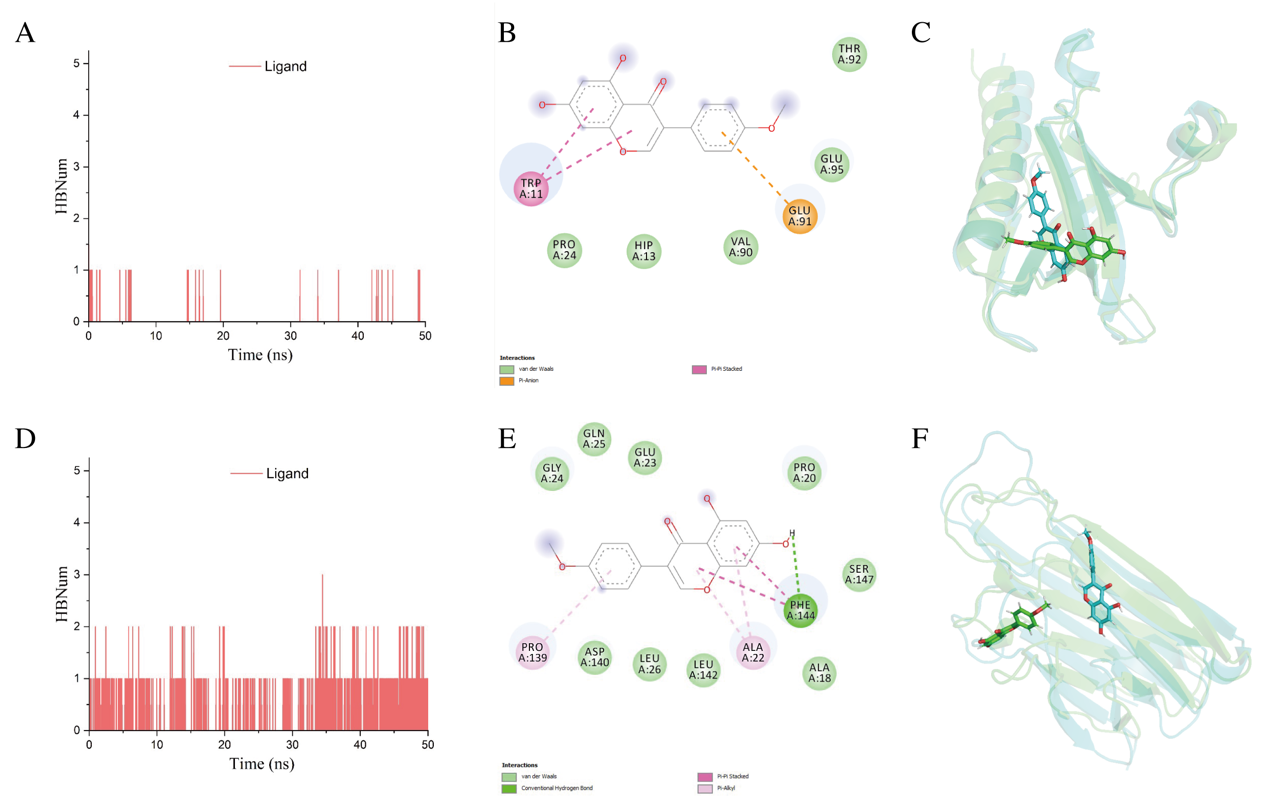


**Supplementary fig. 4 Molecular Dynamics Simulation.** A: Hydrogen bonds between AKT1 and ligands by molecular dynamics simulation; B: The molecular docking models of biochanin A with AKT1; C: Initial and final structure of the Align. D: Hydrogen bonds between TNF-α and ligands by molecular dynamics simulation; E: The molecular docking models of biochanin A with TNF-α; F: Initial and final structure of the Align.
